# Epidemiological patterns of influenza viruses among severe acute respiratory infection patients in Burkina Faso, 2016-2019: a surveillance study

**DOI:** 10.11604/pamj.2025.52.98.43862

**Published:** 2025-11-05

**Authors:** Moussa Lingani, Assana Cissé, Abdoul Kader Ilboudo, Brice Bicaba, Issaka Yaméogo, Zékiba Tarnagada

**Affiliations:** 1 Laboratoire National de Référence-Grippes, Institut de Recherche en Sciences de la Santé, Ouagadougou, Burkina Faso,; 2 Unité de Recherche Clinique de Nanoro, Institut de Recherche en Sciences de la Santé, Nanoro, Burkina Faso,; 3 One Health Association, Ouagadougou, Burkina Faso,; 4 Direction de la Protection de la Santé de la Population, Ouagadougou, Burkina Faso

**Keywords:** Burkina Faso, influenza, severe acute respiratory infections, surveillance

## Abstract

**Introduction:**

influenza viruses cause acute respiratory infections; however, their importance among severe cases remains poorly documented in Sub-Saharan Africa. This study aimed to describe the burden of influenza among inpatients admitted for severe acute respiratory infections (SARI) in Burkina Faso.

**Methods:**

a surveillance study was conducted between 2016 and 2019 by the National Influenza Reference Laboratory (NIRL). Inpatients admitted for SARI from four sentinel surveillance sites were included and tested for influenza using a real-time reverse transcription-polymerase chain reaction (rRT-PCR) with the Fast Track Diagnostics (FTD-33) Kits. Positive samples to influenza virus type A or B were further subtyped using the CDC Primers, probes, and protocols. Descriptive analysis was used to assess the prevalence of influenza according to participants' medical and sociodemographic characteristics using the R statistical package. A p-value less than 0.05 was considered statistically significant.

**Results:**

overall, the prevalence of influenza was 20.1% (310/1541). Influenza virus type-specific prevalence was 13.0% (200/1,541), 6.1% (94/1,541), and 1.0% (16/1,541) for type A, B, and C viruses, respectively. Among the cases, type A virus was predominant with 64.4% (52.4% for A(H1N1)pmd09 and 12.0% for A(H3N2)) and type B virus with 30.4% (21.7% for B/Victoria and 8.1% for B/Yamagata). Three waves of increased transmission were observed during the study period, and each was dominated by a specific virus subtype. The distribution of cases according to sociodemographic characteristics showed that children aged 1-4 years were mostly affected (60%), mainly with type A virus (36.9%), followed by infants below 1 year of age (21%), also with type A virus (15.2%). Virus circulation occurred year-round, and transmission peaks occurred during the cold and dry seasons.

**Conclusion:**

type A influenza virus was predominant, especially among children under five years old. Prioritizing vaccination among preschool-aged children, particularly during the seasonal peaks, might yield the most public health impact.

## Introduction

Despite being vaccine-preventable, influenza epidemics still result in substantial disease burdens worldwide, particularly in low-income countries [1]. Globally, three to five million severe cases and between 290,000 and 650,000 deaths occur annually [2]. Systematic reviews showed that the highest hospitalization rates occur in people aged 65 years or older [3] and children younger than five years of age [4]. Influenza prevalence, particularly epidemic influenza A and B, depends on several factors, and their distributions vary across seasons, geographic regions, and population demographics [5-7]. Today, vaccination remains the primary intervention to prevent influenza and its complications [8,9]. However, vaccination timing is critical considering that vaccine-induced immunity wanes over seasons, as well as a variation of their antigenic compositions [10-12]. Thus, efficiently deploying the vaccines is critical to limit the disease burden, and the WHO recommends annual vaccination using inactivated influenza strains [2,13]. In addition, the vaccination rates vary considerably across the globe, and in 2014, 74% of upper-middle-income countries had an influenza vaccination policy. In contrast, only 37% of lower-middle-income countries and 3% of low-income countries had an influenza vaccination policy [14]. The scarcity of epidemiological data to inform decision-making represents one of the major challenges to expanding preventive measures; thus, detailed data are needed for the development and implementation of targeted interventions [15-17].

In Burkina Faso, surveillance for influenza-like illnesses (ILI, history of fever or measured temperature ≥ 38°C, and cough, with onset within the last ten days ) [18] and severe acute respiratory infections (SARI, history of fever or measured temperature of ≥ 38°C, cough within the last ten days, and hospitalization) [18] was established in 2010 and coordinated by the Ministry of Health (MoH). The first results over the period 2010-2012 showed that 6.6% of ILI patients were confirmed for influenza, and for the period 2013-2015, 14.8% of ILI patients were confirmed for influenza (58.5% influenza A and 41.5% influenza B) [19]. Over 2013-2015, similar findings were reported with A and B viruses reported in 15.1% (112/743) of ILI patients [20,21]. Although these few studies implemented in the urban areas provided useful information on the circulation of influenza viruses in the country, they did not explore their patterns among SARI cases [19-21]. This is particularly noticeable in rural areas where the living conditions further expose populations to acute respiratory infections, thus hampering proper decision-making [19-21]. For adequate disease control measures, detailed virological and epidemiological information is needed. This study aimed to provide updated information on the epidemiological patterns of influenza among severe acute respiratory infection cases in Burkina Faso. General objective: the study's general objective was to describe the epidemiological patterns of influenza viruses´ circulation among inpatients admitted for severe acute respiratory infections (SARI) in Burkina Faso from 2016 to 2019. Specific objectives were to: i) assess the magnitude of influenza viruses´ types and subtypes among inpatients admitted for SARI in four referral hospitals in Burkina Faso; ii) assess the proportions of each virus type/subtype among inpatients diagnosed for influenza during the study period; iii) analyze the temporal trends of the transmission according to seasonality and socio-demographic characteristics.

## Methods

**Study design:** this was a surveillance study implemented by the National Influenza Reference Laboratory (NIRL) between 2016 and 2019 in Burkina Faso. Data were collected prospectively for a descriptive analysis of influenza patterns.

**Study settings:** the data were collected within four referral hospitals: the teaching hospital of Bogodogo in Ouagadougou, the capital city; the District Hospital of Boussé in the “Plateau Central” region; the District Hospital of Houndé in the “Hauts Bassins” region; and the District Hospital of Kongoussi in the “Centre Nord” region. These sites were selected based on several criteria including their geographic representation, the high number of patients, and the availability of refrigerators (+2 to +4°C) for biological specimens´ storage.

**Participants and sample size:** cases were included using the World Health Organization SARI case definition of 2014 (history of fever or measured temperature ≥ 38°C and cough and/or sore throat, onset within the last 10 days, and requiring hospitalization) [18]. The minimum sample size was 652 participants based on the prevalence from a preliminary study [20], and calculated using the Cochran formula


$$\left(N=\frac{Z^{2}P\left(1-P\right)}{I^{2}}\right)$$

where N is the sample size, Z is the z-score that corresponds to the 95% confidence interval (1.96), P is the expected proportion, and I is the margin of error set at 2%. All suspected cases were systematically included if they fulfilled the definition of SARI during the entire study period and consented to participate.

**Variables:** the collected variables included patients´ socio-demographic characteristics (sex, age, residency), clinical symptoms, date of onset of illness, vaccination status, and sample collection date. In addition, information related to the health center of origin, the year of sampling, the setting of residence (rural or urban), the outcome of the hospitalization (cured, in treatment, or death), and the results of the PCR testing were also collected.

**Data source and collection:** health workers from each sentinel site received training on respiratory symptoms screening, specimens´ collection, storage, and transportation. For each patient identified with a severe acute respiratory infection, data were extracted using a standardized questionnaire from patient´s medical records. Information not available in the medical records was obtained by direct interview with the patients or with the authorized representative or from the health booklets.

**Laboratory testing:** for a patient included, nasopharyngeal (NP) and/or oropharyngeal (OP) specimens were collected within 24 hours of hospitalization and placed into Universal Transport Media (Copan Diagnostics). After collection, the specimen was temporarily refrigerated on-sites before shipment in cold packs to the national reference laboratory (NIRL) within 72 hours of collection for analysis or long-term storage in minus 80 degrees centigrade freezers [22]. Briefly, total nucleic acid (TNA) was extracted from 400 μL of Universal Transport Media containing the specimens and eluted into 110 μL using the EZ1 Advanced XL Instrument with the EZ1 Mini Viral 2.0 Kit (Qiagen) following the manufacturer's instructions. Detailed laboratory procedures are described elsewhere [23]. The TNA extracts were subsequently screened to detect respiratory pathogens using the FTD-33 multiplex rRT-PCR according to the manufacturer's instructions [24]. The current report focused on influenza. Specimens tested positive for influenza A and B viruses were subtyped using singleplex rRT-PCR methods from the Centers for Disease Control and Prevention (CDC) influenza Division (Atlanta, GA, USA) [23]. Specifically, specimens that tested positive for influenza A virus were further tested for influenza A virus subtype detection using an influenza A(H3/H1pdm09) panel. An influenza B lineage genotyping panel including B/Victoria and B/Yamagata primers and probes was used for samples that tested positive for B viruses. For each reaction, mix a total of 5 μL of total nucleic acid, 5 μL of nuclease-free water, 0.5 μL of each primer (forward and reverse) and probe, 12.5 μL of 2X AgPath-IDTM One-Step RT-PCR buffer, and 1 μL of 25X AgPath-IDTM One-Step RT-PCR enzyme mix (Thermo Fisher Scientific) was used. Reaction mixtures containing a no-template control (negative control), extraction control, human specimen control, and pooled influenza-positive control were included for each singleplex reaction mix. The rRT-PCR testing was performed at a final volume of 25 μL on the Applied Biosystems 7500 Real-Time PCR Instrument with the following cycling conditions: 50°C for 30 minutes, 95°C for 10 minutes, 40 cycles of 95°C for 15 seconds, and 55°C for 30 seconds. Any assay or specimen with a control result deviating from the expected result was retested [23].

**Minimizing potential biases:** the quality of medical record archiving practices was not consistent across health facilities, and paper-based records are still the standard practice. Some medical records were unable to be located. To minimize biases, all cases were systematically included to address selection biases, and the PCR diagnostic approach was used for case ascertainment biases.

**Data management and analysis:** statistical analysis was conducted using R (R Core Team, 2021), RStudio (RStudio Team, 2023), and the Tidyverse package (Wickham, 2017). We considered descriptive analyses, which comprised assessing frequency distributions and proportions for each categorical variable. Graphs were plotted using Excel, and Chi-Square or Fisher´s exact test for categorical variables was used for group comparisons. Age was categorized as < 1 year, 1-4 years, 5-18 years, and 19 + years. For result interpretation, p-values < 0.05 were statistically significant. Missing values were included as numbers in tables but were not considered in calculations.

**Ethics approval, consent to participate:** as this sentinel surveillance was a public health program implemented by the Burkina Faso Ministry of Health, ethical approval was unnecessary. However, all included patients provided informed consent in accordance with Helsinki recommendations.

## Results

**Demographic characteristics:** of the 1,541 participants admitted in the four sites for SARI during the study period, 56.3% were male, and nearly half of them (56.7%) were from the rural areas. The under-five-year-old children represented nearly 85% of the study population, and flu vaccination rates were below 1% (11/1,530). The overall death rate neared 2% of the study population (Table 1).

**Table 1:** demographic characteristics of patients with SARI, 2016-2019 in Burkina Faso

Characteristics	Number	Percentage
**Sex**		
Female	673	43.7
Male	868	56.3
**Age group (in years)**		
< 1	503	32.7
1-4	807	52.5
5-18	109	7.1
19-+	118	7.7
Missing *	4	-
**Health center**		
Bogodogo	378	24.5
Houndé	469	30.4
Kongoussi	466	30.2
Boussé	228	14.8
**Year of sampling**		
2016	53	3.4
2017	605	39.3
2018	748	48.5
2019	135	8.8
**Residency**		
urban	668	43.3
Rural	873	56.7
**Flu vaccination**		
Yes	11	0.7
No	1519	99.3
Missing*	11	-
**Outcome**		
Cured	1177	81.6
Death	27	1.9
In treatment	239	16.6
Missing*	98	-

* Missing values were not included in calculations, but only presented as a number; SARI: severe acute respiratory infection

**Etiological characteristics:** the overall prevalence of influenza (type A, type B or type C) was 20.1% (310/1,541). The prevalence was 13.0% (200/1,541) for influenza A virus, 6.1% (94/1,541) for influenza B virus, and 1.0% (16/1,541) for influenza C virus. According to influenza subtyped or lineage, the prevalence was 10.6% (163/1,541) for A(H1N1)pdm09, 2.4% (37/1,541) for seasonal A(H3N2), 4.3% (67/1,541) for B Victoria, and 1.6% (25/1,541) for B lineage Yamagata (Table 2). Detected influenza viruses were predominantly of type A (64.4%) composed of 52.4% of A(H1N1)pdm09 and 12% of A(H3N2). Type B viruses were the second most prevalent (30.4%), with 21.7% of B/Victoria, and 8.1% of B/Yamagata (Table 3).

**Table 2:** influenza virus type-specific prevalence among 1,541 SARI inpatients population according to the influenza virus type and subtype (2016-2019), Burkina Faso

Influenza type	Influenza subtype	Prevalence (%)
Influenza A		13.0
;	Influenza A (H1N1) pdm09	10.6
	Influenza A (H3N2)	2.4
Influenza B	;	6.1
	Influenza B/Victoria	4.3
	Influenza B/Yamagata	1.6
Influenza C	-	1.0
At least one type	A, B, C	20.1

SARI**:** severe acute respiratory infection

**Table 3:** distribution of the types and subtypes of influenza viruses detected according to patients’ age-groups

	Influenza types and subtypes n (%)						
Age groups	A(H1N1) pdm09	A(H3N2)	B/Victoria	B/Yamagata	B/Unsubtyped	Influenza C	Total
< 1 year	39 (12.6)	8 (2.6)	11 (0.7)	3 (0.2)	0 (0.0)	4 (0.3)	65 (21.0)
1-4 years	89 (28.8)	25 (8.1)	44 2.9)	14 (0.9)	2 (0.1)	11 (0.7)	185 (59.9)
5-18 years	17 (5.5)	4 (1.3)	8 (0.5)	5 (0.3)	0 (0.0)	0 (0.0)	34 (11.0)
≥ 19 years	17 (5.5)	0 (0.0)	4 (0.3)	3 (0.2)	0 (0.0)	1 (0.1)	25 (8.1)
Total	162 (52.4)	37 (12.0)	67 (21.7)	25 (8.1)	2 (0.6)	16 (5.2)	309 (100.0)

**Influenza distribution according to socio-demographic characteristics:** regarding the distribution of influenza virus types according to patients´ sociodemographic characteristics, children aged 1-4 years old were the most represented (59.9%), followed by those less than one year of age (21.0%). Children and adolescents aged 5 to 18 years represented 11% of the cases, while 8.1% of the influenza cases affected adult populations (Table 3).

**Figure 1:**
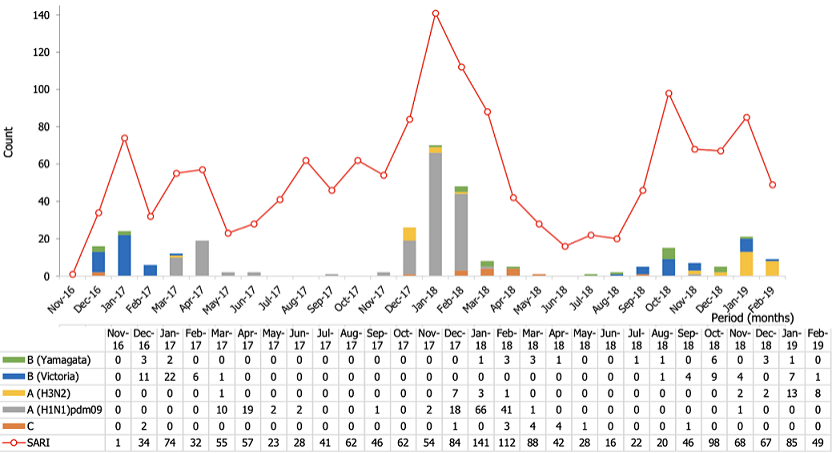
monthly trend of SARI cases and confirmed influenza types, subtyped and lineage from 2016 to 2019, Burkina Faso

**Influenza distribution according to the seasonality:** the temporal distribution of influenza cases showed that overall, three waves of increased transmission were observed during the study period, with each wave led by a different subtype of the virus. The high transmission periods overlapped with the cold and dry seasons, and the highest peak occurred between December 2017 and March 2018 and was made of A(H1N1) pdm09 subtype (Figure 1).

## Discussion

From 2016 to 2019, one out of five patients with severe acute respiratory infection (SARI) was confirmed positive for influenza viruses. This prevalence was higher than the 6.6% influenza-confirmed SARI cases previously reported by the surveillance system in Burkina Faso in 2015 [20]. The observed difference could be related to the surveillance setting and the influenza types tested. Indeed, in addition to the previously reported type/lineage of influenza by the NIRL's first report, the current study included the results for influenza C testing and data from the rural area, which provide a much broader picture of the magnitude of influenza among SARI cases. Lower figures were reported in other African countries, such as Kenya, with approximately 10% influenza-confirmed SARI cases [25]. The climatic patterns are reported to influence influenza transmission [26-28]. Indeed, studies suggested that high ambient temperature and a drop in temperature and their interaction increase the risk of infection with influenza activities peaking during the cold-dry seasons [26]. This trend was supported by different prevalence studies conducted in the Middle East (with 18% in Pakistan and 59% in Yemen) [29,30]. This implies that studies are needed in each setting to describe the patterns and evolution of the influenza epidemics to inform decision-making, and prevention policies should be contextualized to maximize their benefits. Influenza A was the most frequent type, especially the A(H1N1) pdm09 subtype. A different trend was reported by the NIRL in 2015, with A(H3N2) the most represented subtype among both ILI and SARI patients (69.1% for A(H3N2) versus 30.9% for A(H1N1) pdm09) [20]. The small sample size in the first report could have also understated the magnitude of A(H1N1) pdm09, suggesting a larger sample size for a more accurate assessment. A difference in subtype periodicity could also explain this shifting [31,32], highlighting the importance of continuous surveillance and the development of subtype-specific prevention and control measures.

This shifting trend was also reported for the influenza B virus, with no case of B/Victoria lineage reported in 2015, while cases for B/Yamagata lineage were reported. In the current study, B/Victoria lineage was the most reported, followed by cases of B/Yamagata lineage [20]. Continuous influenza surveillance would be suggested to update the antigenic composition of the vaccine for a better public health response. Young children, mainly preschool-age children (1-4 years), were the most represented among SARI cases and the most at risk of influenza. Studies in Burkina Faso and other countries in Africa reported similar findings among children under five years of age were the most affected with influenza among SARI and ILI patients [19-21,33-35]. This suggests that preventive interventions, particularly vaccination, would be more beneficial if preschool-age children were mainly targeted. Although substantial variations across years of surveillance are reported, influenza epidemics mostly appear during the dry and cold seasons (November-March), corresponding to the Harmattan season (colder, dry, windy season). The timing of SARI and influenza overlaps with peak transmission seasons, occurring during the cold and dry periods and during the period of rapid drop in average temperatures [36-38]. This trend is widely reported and suggests that seasonal characteristics should be considered in developing and implementing preventive interventions. The vaccination rate in this report was low as expected. Vaccination for influenza is not yet a public health intervention in Burkina Faso and adequate decision making will require a proper understanding of the magnitude of the infection among vulnerable population, the seasonal circulation of influenza viruses, the type and subtypes of circulating viruses, as well as the timing of epidemics, peak transmissions and seasonal variation in intensity of influenza activity to help design and implement the more beneficial policies [8,9].

**Study limitations and interpretation:** substantial limitations are worth consideration for this study's results interpretation, mainly due to numerous challenges to the influenza surveillance system in the country. Indeed, the quality of medical records is highly impacted by the disease burden on health practitioners, and due to current practices in health facilities of paper-based recording data. Thus, several medical records could not be located, and some available records could not be exploited. In addition, the exact cause of death was not captured to distinguish death due to influenza or a worsening of an underlying condition. The study, however, presents the magnitude and variation of influenza among patients hospitalized for acute respiratory infections, which might be useful for decision-making.

## Conclusion

The study findings showed that influenza virus substantially contributes to the etiologies of SARI in Burkina Faso, particularly among children under five years old. Current data support influenza vaccination of preschool-age children during the cold and dry seasons. The surveillance system should be reinforced to provide detailed information to refine vaccine recommendations. Additional preventive interventions are required to mitigate influenza contribution to SARI occurrence in the country.

### What is known about this topic

- Influenza is a severe public health problem in Burkina Faso;
- The distribution of influenza among acute respiratory infection (ARI) cases is known.

### What this study adds

- Detailed information on the distribution of influenza among severe acute respiratory infection (SARI) cases in Burkina Faso;
- Detailed data on year-round circulation of influenza viruses among SARI cases in Burkina Faso is available;
- Preschool-age children bear the highest burden of influenza among SARI cases.
